# Complete genome sequence of SARS-CoV-2 isolated from a dog in Kazakhstan

**DOI:** 10.1128/mra.00030-25

**Published:** 2025-08-12

**Authors:** Ainur Nurpeisova, Sandugash Sadikaliyeva, Zhandos Abay, Kuanysh Jekebekov, Kamshat Shorayeva, Syrym Kopeyev, Nurkuisa Rametov, Elina Kalimolda, Sabina Moldagulova, Yeraly Shayakhmetov, Alisher Omurtay, Kulyaisan Sultankulova, Nurlan Kozhabergenov, Bekbolat Usserbayev, Asankadyr Zhunushov, Aidyn Kydyrmanov, Yuriy Skiba, Bolat Yespembetov, Sergazy Nurabayev, Lespek Kutumbetov, Balzhan Myrzakhmetova, Berik Khairullin, Hansang Yoo, Aslan Kerimbayev, Markhabat Kassenov, Kunsulu Zakarya

**Affiliations:** 1 Research Institute for Biological Safety Problems 375535 , Guardeyskiy, Kazakhstan; 2 Institute of Biotechnology, National Academy of Sciences 185179, Bishkek, Kyrgyz Republic; 3 Research and Production Center of Microbiology and Virology 374530 , Almaty, Kazakhstan; 4 Almaty Branch of the National Center for Biotechnology, Almaty, Kazakhstan; 5 MVA Group Scientific-Research Production Center Ltd., Almaty, Kazakhstan; 6 College of Veterinary Medicine, Seoul National University, Seoul, Republic of Korea; Queens College Department of Biology, Queens, New York, USA

**Keywords:** genome sequence, SARS-CoV-2, dog, next generation sequencing

## Abstract

We report the complete genome sequence of SARS-CoV-2 isolated from a rectal swab of a dog in Almaty, Kazakhstan. Phylogenetic analysis identified the isolate as an early divergence of lineage B. These findings contribute to understanding the zoonotic potential of SARS-CoV-2 and its implications for public health.

## ANNOUNCEMENT

The ongoing SARS-CoV-2 pandemic has underscored the importance of surveillance for zoonotic reservoirs and transmission pathways. SARS-CoV-2 belongs to the family *Coronaviridae*, subfamily *Orthocoronavirinae*, genus *Betacoronavirus*, subgenus *Sarbecovirus*, and species *Betacoronavirus pandemicum* (1, 2). In this context, companion animals such as dogs and cats have raised concern as potential hosts (3). To explore this, we conducted a study in Kazakhstan to monitor coronaviruses in domestic animals.

A complete genome of SARS-CoV-2 was obtained from a virus isolated from a domestic dog in Almaty, Kazakhstan. The isolate was recovered from a rectal swab and identified as a member of lineage B, closely related to the Delta variant (B.1.617.2). This report provides genomic and phylogenetic data to support the ongoing SARS-CoV-2 surveillance in animal hosts.

A rectal swab was collected from a pet dog presenting mild respiratory symptoms in 2022. The RNA was extracted using the QIAamp Viral RNA Mini Kit (Qiagen, Germany) and tested for SARS-CoV-2 using the ALSENSE-SARS-CoV-2 RT-qPCR assay (Al-Sense, Kazakhstan), targeting ORF1ab and N genes. Sample collection was approved by the Institutional Ethics Committee of the Research Institute for Biological Safety Problems (protocol #5, 29 November 2021).

Virus isolation was performed on Vero cells (ATCC CRL-1586) by carrying out three blind passages under standard conditions. Cytopathic effects appeared by the third passage. Clarified and concentrated supernatants were used for RNA extraction, and cDNA synthesis was carried out using the Ion Torrent NGS Reverse Transcription Kit. Fragmentation and adapter ligation were performed with the Ion Plus Fragment Library Kit, and amplified libraries were purified and quantified using standard Ion Torrent protocols. Size-selected DNA fragments (350–500 bp) were sequenced on the Ion GeneStudio S5 system using an Ion 530 Chip.

Read processing, trimming, and reference-based assembly (MN908947.3) were completed using Torrent Suite Software v5.12 and UGENE v52. No host genome filtering was performed prior to analysis, as the virus was propagated in Vero cells (originating from *Chlorocebus sabaeus*). A total of 3,217,609 high-quality reads (average length: 310 bp) were generated and aligned to the SARS-CoV-2 reference genome (GenBank: MN908947.3), resulting in 100% genome coverage. The complete genome of the isolate SARS-CoV-2/Canis lupus familiaris/KAZ/CCoV_Almaty_KZ_2022/2022 is 29,903 bp in length with a GC content of 38.0% and an average sequencing depth of 2,530×.

**Fig 1:**
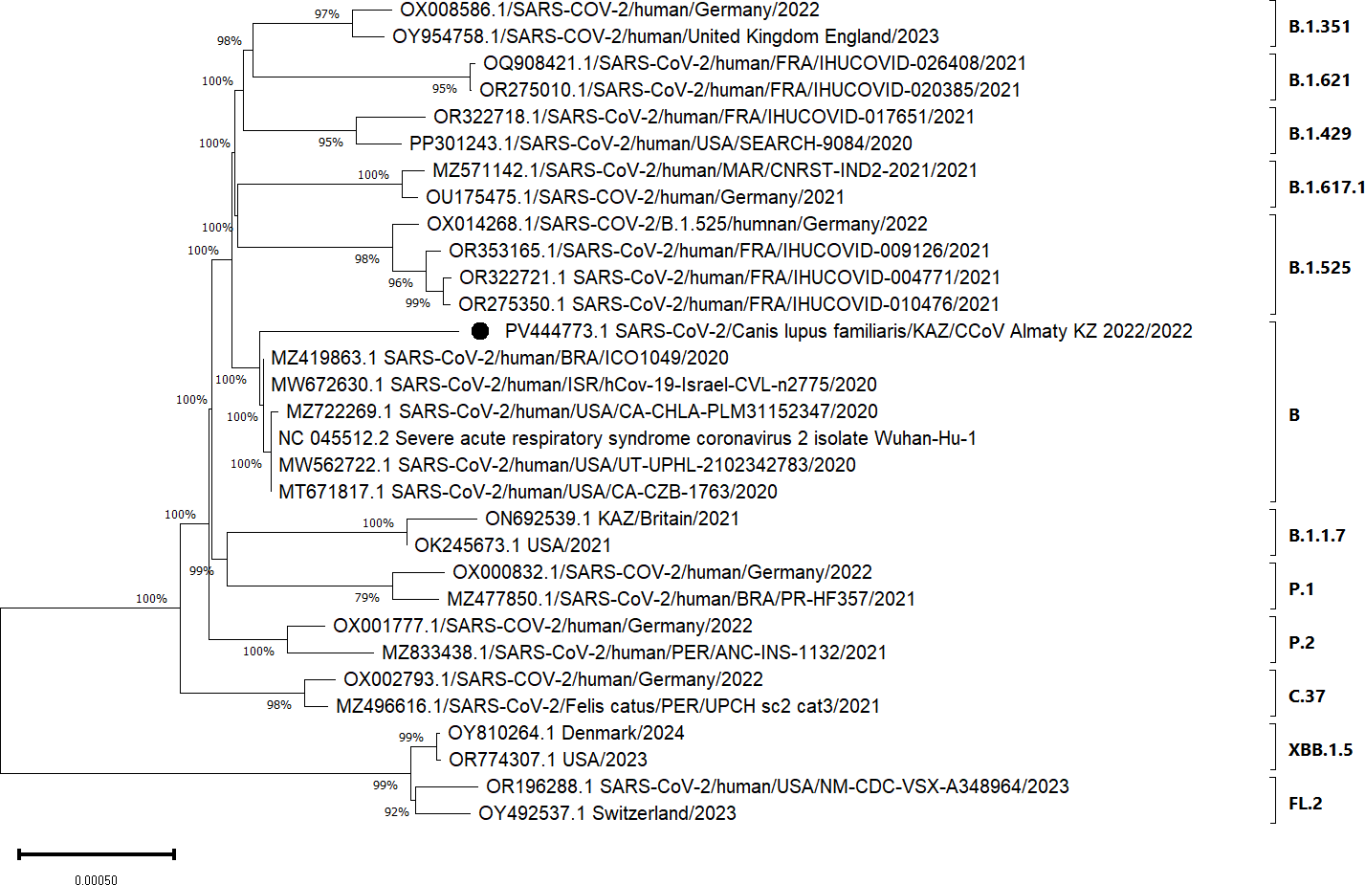
Phylogenetic analysis of the complete genome of SARS-CoV-2/Canis lupus familiaris/KAZ/CCoV_Almaty_KZ_2022/2022 and 30 global SARS-CoV-2 strains representing different lineages. The sequence obtained in this study is marked with a black dot. The phylogenetic tree was constructed using the Neighbor-Joining method (4) implemented in MEGA11 (5). Evolutionary distances were calculated using the Tamura–Nei model (6) and are expressed as the number of base substitutions per site. All ambiguous positions were removed using pairwise deletion. Multiple sequence alignment was performed with the MUSCLE algorithm under default parameters. The analysis included 31 full-length nucleotide sequences. Reference sequences were obtained from GenBank (accession numbers shown in the phylogenetic tree). Bootstrap values were calculated with 1,000 replicates and are shown at the corresponding branches.

Phylogenetic analysis placed the isolate (SARS-CoV-2/Canis lupus familiaris/KAZ/CCoV_Almaty_KZ_2022/2022) within lineage B and in close proximity to Delta variant strains (B.1.617.2) circulating in 2021–2022 (Fig. 1).

The isolate exhibits multiple mutations, including 10 in the spike (S) gene and 8 in ORF1ab, notably S:D614G and ORF1ab:K1037T, suggesting early divergence within lineage B (Table 1).

**TABLE 1:** Comparison of amino acid substitutions between the Wuhan-Hu-1 and KAZ/CCoV_Amaty_KZ_2022/2022 strains

Protein	Wuhan-Hu-1		KAZ/CCoV_Amaty_KZ_2022/2022		Amino acid substitution
	Position	Variant	Position	Variant	
ORF1ab	5,829	A	C	5,829	K1037T
	9,749	A	G	9,749	K399E
	9,867	T	G	9,867	L438R
	12,904	T	C	12,904	C73C
	15,017	C	T	15,017	A517V
	16,722	A	G	16,722	E162E
	20,759	C	T	20,759	A34V
	21,446	A	G	21,446	K263R
S	21,642	C	T	21,642	A27V
	21,646	C	T	21,646	Y28Y
	21,648	C	T	21,648	T29I
	21,784	T	A	21,784	N74K
	21,789	C	T	21,789	T76I
	21,846	C	T	21,846	T95I
	22,036	A	C	22,036	R158S
	23,014	A	C	23,014	E484D
	23,403	A	G	23,403	D614G
	23,520	C	T	23,520	A653V
	23,751	C	T	23,751	S730F
ORF3a	25,688	C	T	25,688	A99V
	26,110	C	T	26,110	P240S
M	26,895	C	T	26,895	H125Y
3′ UTR	27,389	C	T	27,389	27389
ORF7a	27,542	C	A	27,542	A50D
	27,630	C	T	27,630	A79A
	27,667	G	A	27,667	E92K
	27,739	C	T	27,739	L116F

According to the phylogenetic analysis based on the complete genome (Fig. 1), the strain is genetically closest to human isolates from Europe and Asia, including strains from Germany, France, and the United Kingdom, showing up to 99% nucleotide identity (based on the GenBank accession numbers shown in the phylogenetic tree). Several unique amino acid substitutions were also detected (Table 1), consistent with patterns observed in other animal-derived SARS-CoV-2 isolates.

## Data Availability

The complete genome sequence of SARS-CoV-2/Canis lupus familiaris/KAZ/CCoV_Almaty_KZ_2022/2022 is available at GenBank under the accession number PV444773. The raw sequence reads were deposited under BioProject: PRJNA1257888 and SRA: SRR33915362.
